# Clinical Images: Sevoflurane‐induced periostitis

**DOI:** 10.1002/art.43435

**Published:** 2026-02-06

**Authors:** Christian Beyer, Marion Ganslmayer, Richard Strauß, Bernhard Manger

**Affiliations:** ^1^ Department of Medicine 1 Friedrich‐Alexander‐University Erlangen‐Nuremberg Erlangen Germany; ^2^ Department of Medicine 3 Friedrich‐Alexander‐University Erlangen‐Nuremberg Erlangen Germany

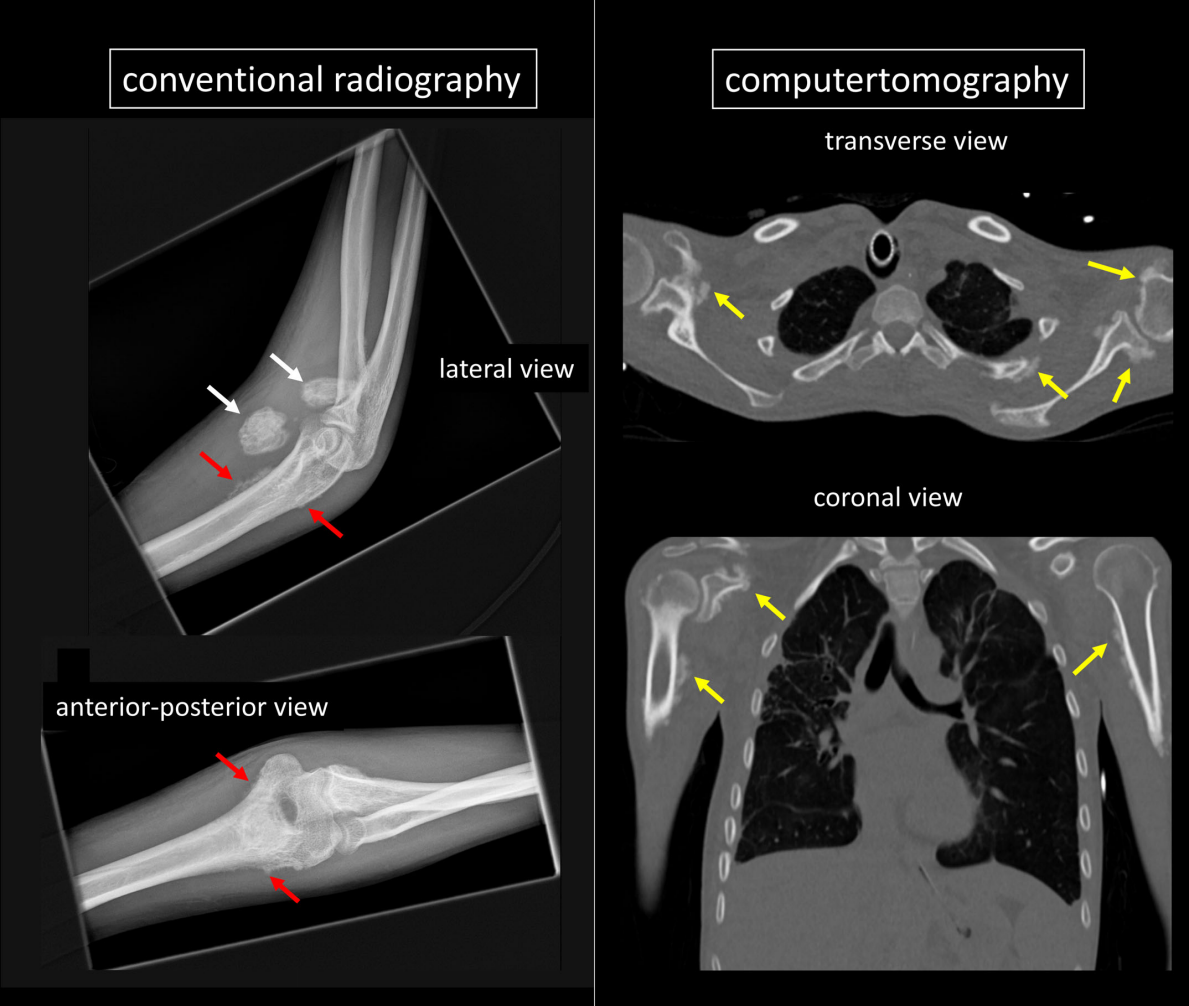

A 25‐year‐old patient was treated with the volatile anesthetic sevoflurane for approximately three months due to agitation unresponsive to intravenous anesthetics. The patient had septic multiorgan failure resulting from a Bartholin abscess and COVID‐19 pneumonia with bacterial superinfection and empyema. Chronic medical conditions included GAD65 antibody–positive autoimmune encephalitis, temporal lobe epilepsy, and Ig deficiency syndrome. The patient developed clinical signs of monoarthritis at the right elbow, raising suspicion for septic or autoimmune arthritis. Arthrosonography revealed severe pericapsulitis without distinct synovitis and joint effusion. Arthrocentesis yielded no fluid. X‐ray imaging demonstrated signs of calcifying periostitis (red arrows), amorphous calcifications at the medial aspect of the joint capsule (white arrows), and altered trabeculation in the juxtaarticular distal humerus. A subsequent chest computed tomography identified periostitis with new bone formation of ribs two to eight bilaterally, both scapulae, and both proximal humeri (yellow arrows). Fluoride levels were elevated in serum (1.62 mg/L; normal <0.1 mg/L) and urine (47.0 mg/L; normal <1.0 mg/L). This led to a diagnosis of sevoflurane‐induced skeletal fluorosis. Three weeks after discontinuing sevoflurane, clinical and ultrasound signs regressed, and fluoride levels in serum (0.24 mg/L) and urine (1.7 mg/L) dropped. Unfortunately, the patient died soon thereafter due to refractory septic shock. Approximately 5% of inhaled sevoflurane are metabolized to hexafluoroisopropanol with release of inorganic fluoride and carbon dioxide.^1^ Although skeletal fluorosis from environmental exposure^2^ and long‐term voriconazole exposure^3^ is recognized, to our knowledge, this is the first reported case associated with volatile anesthetics. Informed consent could not be obtained because the patient was deceased. All efforts were made to ensure that no personally identifiable information was disclosed.

## Supporting information


**Disclosure Form**:
